# The inverse associations of glycine and histidine in diet with hyperlipidemia and hypertension

**DOI:** 10.1186/s12937-024-01005-4

**Published:** 2024-08-22

**Authors:** Mohammad Haroon Rahemi, Yuting Zhang, Zican Li, Dongwei Guan, Defang Li, Hongxin Fu, Jiaying Yu, Junrong Lu, Cheng Wang, Rennan Feng

**Affiliations:** 1 Department of Nutrition and Food Hygiene, School of Public Health, Harbin Medical University, 157 Baojian Road, Nan gang District, Harbin, 150081 Heilongjiang China; 2 Key Laboratory of Precision Nutrition and Health of Ministry of Education, School of Public Health, Harbin Medical University, Harbin, 150081 Heilongjiang China; 3 Department of Interventional Radiology, Harbin Medical University Cancer Hospital, Harbin, 150081 Heilongjiang China; 4 Department of Environmental Hygiene, School of Public Health, Harbin Medical University, Harbin, 150081 Heilongjiang China

**Keywords:** Amino acids, Diet, Hyperlipidemia, Hypertension, Nutrition surveys

## Abstract

**Background:**

Amino acids are crucial for nutrition and metabolism, regulating metabolic pathways and activities vital to organismal health and stability. Glycine and histidine act as potent antioxidants and anti-inflammatory agents; however, limited knowledge exists regarding the associations between these amino acids and hyperlipidemia and hypertension. The purpose of this study is to investigate the relationship between dietary glycine and histidine, and hyperlipidemia and hypertension.

**Methods:**

This population-based cross-sectional study evaluated the influence of dietary glycine and histidine, as well as their combined effect, on hyperlipidemia and hypertension in Chinese adults participating in the Nutrition Health Atlas Project (NHAP). General characteristics were acquired using a verified Internet-based Dietary Questionnaire for the Chinese. Binary logistic regression, along with gender, age groups, and median energy intake subgroup analyses, was employed to investigate the associations between dietary glycine and histidine and hyperlipidemia and hypertension. A sensitivity analysis was conducted to assess the impact of excluding individuals who smoke and consume alcohol on the results.

**Results:**

Based on the study’s findings, 418 out of 1091 cases had hyperlipidemia, whereas 673 had hypertension. A significant inverse relationship was found between dietary glycine, histidine, and glycine + histidine and hyperlipidemia and hypertension. Compared with the 1st and 2nd tertiles, the multivariable-adjusted odd ratios (ORs) (95% confidence intervals) (CIs) of the 3rd tertile of dietary glycine for hyperlipidemia and hypertension were 0.64 (0.49–0.84) (*p* < 0.01) and 0.70 (0.56–0.88) (*p* < 0.001); histidine was 0.63 (0.49–0.82) (*p* < 0.01) and 0.80 (0.64–0.99) (*p* < 0.01); and glycine + histidine was 0.64 (0.49–0.83) (*p* < 0.01) and 0.74 (0.59–0.92) (*p* < 0.001), respectively. High glycine and high histidine (HGHH) intake were negatively associated with hyperlipidemia and hypertension OR (95% CIs) were: 0.71 (0.58–0.88) (*p* < 0.01) and 0.73 (0.61–0.87) (*p* < 0.01), respectively.

**Conclusions:**

Dietary glycine and histidine, as well as their HGHH group, revealed an inverse relationship with hyperlipidemia and hypertension. Further investigations are needed to validate these findings.

**Supplementary Information:**

The online version contains supplementary material available at 10.1186/s12937-024-01005-4.

## Background

Amino acids (AAs) play a vital role in nutrition and metabolism; they act as a crucial precursor for the synthesis of a wide range of important compounds, as well as regulate key metabolic pathways and processes critical to organismal vitality, growth, development, reproduction, and homeostasis [1, 2]. Previous studies have indicated that abnormalities in amino acid metabolism have been associated with a variety of medical ailments, including metabolic diseases such as hyperlipidemia and hypertension [3, 4], as well as cardiovascular diseases [5]. Glycine, a major amino acid in humans, is essential for nutrition and can be obtained either through the diet or produced endogenously in the body from choline, serine, threonine, and hydroxyproline in the liver and kidneys [6]. Moreover, glycine has anti-inflammatory and antioxidant effects; it aids glutathione production, protecting cells from oxidative damage, alcohol-induced liver damage, and oxidized oils, promoting liver recovery, and food toxicity protection [7]. A recent study has reported an inverse relationship between lower glycine levels and an elevated risk of hypertension [8]. Increasing evidence suggests that glycine supplements improve lipid profiles and decrease cholesterol in animal trials. For instance, a study conducted in hypercholesterolemic rats showed that oral supplementation with glycine reduced hepatic cholesterol by 29% and the plasma cholesterol-to-phospholipid ratio by 40% [4, 9]. Histidine is a powerful antioxidant and anti-inflammatory agent that scavenges free radicals, binds divalent metal ions, and resists glycation; food intake, enzymatic breakdown, and urine excretion all influence its levels in the blood [10, 11]. This amino acid is nutritionally vital for mammals, possessing distinct biochemical and physiological characteristics; these properties give a strong theoretical rationale for the use of histidine as a versatile dietary supplement for various health issues [12]. Currently, histidine and histidine-containing peptides are under scrutiny for their effectiveness in preventing ageing-related disorders such as atherosclerosis, neurological disorders (including Alzheimer’s disease), cancer, metabolic syndrome (MetS), and people with obesity [12–14]. Of particular note, histidine has been found to decrease the risk of metabolic and cardiovascular diseases [15]. L-histidine has the ability to decrease blood pressure in spontaneously hypertensive rats via diminishing sympathetic output through the central histamine H3 receptor and enhancing nitric oxide in the rostral ventrolateral medulla [16]. Histidine-containing dipeptide supplementation decreased total cholesterol and triglyceride levels by preventing low-density lipoprotein cholesterol oxidation [17].

Hyperlipidemia and hypertension, essential components of the metabolic syndrome [4], raise the risk of premature death and are major contributors to mortality and disability worldwide [18]. Hyperlipidemia is a prominent risk factor for metabolic ailments; globally, millions of adults suffer from elevated levels of total cholesterol (TC) or triglycerides (TG) [19]. High blood cholesterol accounts for roughly 18% of strokes and 56% of heart attacks around the world [20]. Hypertension, the main cause of mortality and morbidity, affects around 25% of the world’s population [21]. Around 44.7% of Chinese adults between the ages of 35 and 75 suffer from high blood pressure, while nearly 244.5 million Chinese adults being impacted by hypertension [22]. Recent studies have extensively examined the roles of carbohydrates and lipids in several metabolic illnesses, but the roles and significance of dietary glycine and histidine remain unclear. It has been reported that it is still ambiguous whether the plasma level of essential amino acids will be influenced by hyperlipidemia [19]. Despite these, previous studies have demonstrated that a high-protein diet reduces body weight, postprandial glucose levels, hyperlipidemia, hypertension, inflammation, and cardiovascular risk; consequently, reasonable dietary intervention should be effective in preventing metabolic diseases [23–26]. Intriguingly, increasing evidence indicates that glycine and histidine supplementation can be a novel therapy for metabolic diseases; particularly, adding glycine to the diet reduce the concentrations of free fatty acids and triglycerides, while histidine improves both hyperlipidemia and metabolic syndrome [6, 12]. Based on the mentioned conclusive evidence, dietary glycine and histidine may also be linked to improvements in hyperlipidemia and hypertension, but their contribution is not yet fully confirmed. Thus, the objective of this study is to assess the relationship between dietary glycine and histidine and hyperlipidemia and hypertension using data from an internet-based dietary and lifestyle questionnaire for Chinese participants (IDQC, 2014–2019).

## Methods

### Study population

The data utilized in this study were acquired from the NHAP database, accessible at: (http://www.yyjy365.org/nhap/index.php/index/idqc.html), accessed on January 15, 2024 [27]. The NHAP was an exploratory dietary and lifestyle survey conducted in China to evaluate the nutrition and health status of the Chinese population. It was carried out using an IDQC, which served as a tool for evaluating the dietary patterns and lifestyle habits of the Chinese population. In contrast to the previous method, the internet-based dietary questionnaire was a more valuable data collection tool, offering enhanced data quality, cost reduction, and a heightened response rate [28, 29]. A convenient instrument known as the IDQC was previously designed and validated at Harbin Medical University by experts in biostatistics, epidemiology, and nutrition; the reliability and accuracy of the IDQC as an effective tool for assessing dietary intake among Chinese populations have been rigorously confirmed in our previous study [30]. The present investigation encompasses data collected between 2014 and 2019. The IDQC survey consists of four components: basic demographic information, physical examination details, lifestyle factors, and dietary intake patterns. Every participant was allowed to create an account at: www.yyjy365.org/diet. To avoid duplicate registrations, the telephone number of each participant was utilized as their account number. All participants submitted online informed consent, as well as details regarding dietary intake, demographics, and the occurrence of hyperlipidemia and hypertension obtained through the IDQC.

**Fig. 1:**
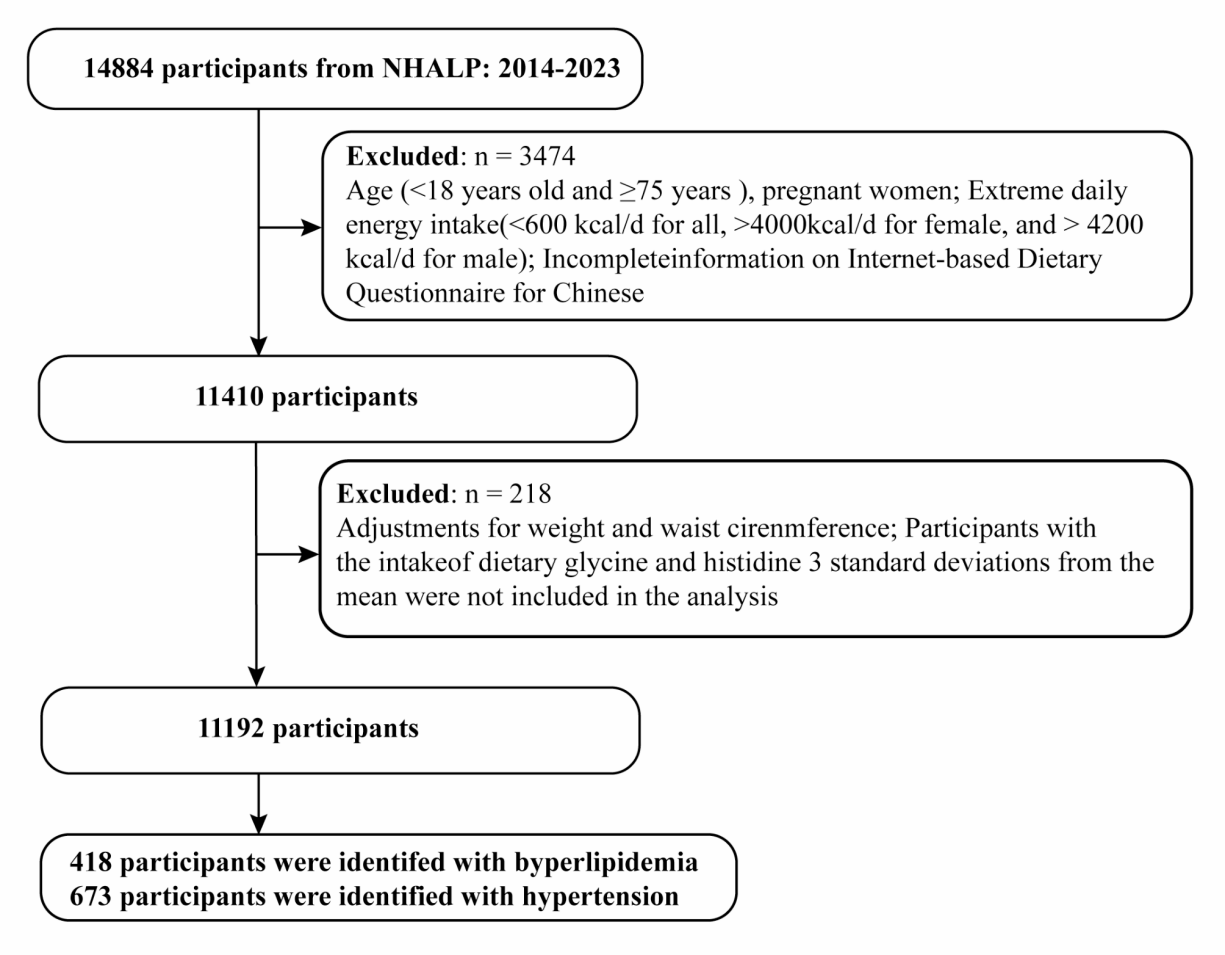
The study participants’ selection flow chart provides a visual representation of the inclusion and exclusion criteria, detailing each step of the selection process and the number of individuals at each stage

A cross-sectional study was carried out on individuals aged 19 to 75 years residing in Heilongjiang province, located in northern China. The exclusion criteria were defined as follows: (a) age below 18 or equal to or above 75; (b) inadequate information provided in the IDQC; (c) extreme daily energy consumption (< 600 kcal/day for both males and females, > 4000 kcal/day for females, and > 4200 kcal/day for males) [24]. A total of 14,884 individuals completed the IDQC questionnaire through the designated website. After applying the exclusion criteria, 11,192 individuals were considered eligible for inclusion in the study (Fig. 1). Out of the respondents surveyed, a total of 418 individuals were found to have high blood cholesterol (hyperlipidemia), while 673 participants were identified as having high blood pressure (hypertension). The Ethics Committee of Harbin Medical University has granted approval for this study. The present study ethical consent number was HMUIRB2019006PRE. All respondents gave informed consent, and the study was carried out in accordance with the ethical guidelines outlined in the Declaration of Helsinki.

### Estimation of dietary nutrient intake

After obtaining informed consent, the participant undergoes a comprehensive face-to-face interview and a detailed physical examination at the nearby health examination facility. Each participant was given the opportunity to complete the IDQC, recalled dietary histories from the recent past (at least four months), and indicated the quantity and frequency of each type of food. One distinguishing feature of IDQC was the presentation of each food item’s weight or volume as a reference to assist participants in making accurate estimations and reducing the chance of recall bias. The daily consumption of all elements was calculated using the China Food Composition Tables to determine the average daily intakes of all nutrients [18, 24, 31]. China Food Composition Tables are a valuable reference book for Chinese nutrition and public health professionals. This book evaluates commonly consumed Chinese foods and provides useful information, including the average nutrient contents (such as energy, macronutrients, trace elements, amino acids, and fatty acids), as assessed by researchers [18, 23, 31]. The combined effect of glycine and histidine (glycine + histidine) was evaluated by summing their intake amounts.

### Demographic, life style and anthropometric information

Demographic and lifestyle data, including age (years), gender (male or female), smoking status (no or yes), alcohol consumption (no or yes), work intensity (light, moderate, severe), monthly income (< 2000yuan, 2000-5000yuan, and > 5000yuan per month), and educational status (under-college, bachelor’s degree, master’s degree, or doctorate) were collected through a self-reported questionnaire. A qualified physician meticulously collected anthropometric details in a dedicated examination room. This process involved precise measurements of weight, height, waist circumference (WC), systolic blood pressure (SBP), and diastolic blood pressure (DBP), as well as BMI kg/m^2^. Body mass index (BMI) was calculated by dividing the weight in kilograms by the square of height in meters (kg/m^2^) [23]. The BMI benchmarks (cut-off points) for Chinese individuals were used as follows: (overweight: 24.0–27.9 kg/m^2^; people with obesity ≥ 28.0 kg/m^2^) [31]. High blood pressure is defined as SBP equal to or exceeding 130 mmHg, or diastolic blood pressure equal to or exceeding 85 mmHg [1]. The existence of hypertension and hyperlipidemia (which is informed by the doctor’s diagnosis) has been identified and confirmed through the use of the IDQC [18, 23].

### Statistical analysis

Continuous variables were displayed as the mean ± standard deviation (SD), whereas categorical variables were exhibited as numerical representations (percentages). The regression residual method was used to adjust the dietary glycine and histidine levels according to the total energy intake, and calculated in milligrams per day (mg/day) [18, 32]. The variations in demographic characteristics between the two groups, classified by median dietary intake of glycine and histidine, were compared among participants with hyperlipidemia and hypertension using one-way analysis of variance (one-way ANOVA). The dietary intake levels of glycine and histidine were categorized into three groups based on their tertile distribution. The reference group for this categorization was the first tertile, which represented the lowest range of dietary intake for these amino acids [33, 34]. The binary logistic regression model was employed, controlling for potential covariates, to calculate the OR and 95% CI, and to investigate the relationship between dietary glycine and histidine and hyperlipidemia and hypertension, respectively. Model 1 was controlled for age and gender; model 2 included covariates from model 1 along with smoking status, alcohol consumption, and work intensity; model 3 incorporated covariates from model 2 in addition to educational status, monthly household income, and BMI. In order to define the range of dietary intake of glycine and histidine for participants, we treated the intake of glycine and histidine in twos, and compared the relationship between the combination of participants in high intake group and low intake group of glycine and histidine and hypertension and hyperlipidemia, so as to evaluate the influence of different levels of dietary intake on the research results. Participants were categorized into four groups for dietary glycine and histidine based on their median dietary levels: the high glycine and low histidine (HGLH) group; the HGHH group; the low glycine and low histidine (LGLH) group; and the low glycine and high histidine (LGHH) group, respectively. Using the LGLH group as the reference group, binary logistic regression was employed in all models to compare the risk of the other groups, respectively. Subgroup analyses were performed across various categories, including males (*N* = 5029), females (*N* = 6163), individuals aged below 60 years (*N* = 9206), those aged 60 years and above (*N* = 1986), participants with energy intake below the median (*N* = 5596), those with energy intake above the median (*N* = 5596). Sensitivity analysis was conducted to exclude smokers and drinkers respectively, and finally non-smokers (*N* = 9170) and non-drinkers (*N* = 9542) were included. The analysis of the study’s data was conducted using R version 4.2.3, developed by the R Foundation for Statistical Computing in Vienna, Austria. Statistical significance was determined by a p for trend of less than 0.05.

## Results

### Demographic characteristics

Based on the findings of this study, among the 11,192 individuals assessed in the NHAP (2014–2019), 1091 participants exhibited the specified outcomes, consisting of 418 instances of hyperlipidemia and 673 instances of hypertension. Compared to participants with dietary glycine and histidine intake smaller than the median, those with intake larger than the median were older, predominantly male, and exhibited a higher prevalence of obesity. Both participants with dietary glycine and histidine exhibited a significant difference in monthly household income, educational status, BMI, alcohol consumption, and work intensity between the two groups. No evident difference in gender, cigarette smoking, and prevalence of hyperlipidemia and hypertension were observed between these two groups (Table 1).

**Table 1:** Selected characteristics of participants by median dietary glycine and histidine intake in the NHAP, 2014–2019

Characteristics	Glycine			Histidine		
	≤ 2637.89	> 2637.89	*P* value	≤ 1464.77	> 1464.77	*P* value
**Number of participants**	5596	5596		5596	5596	
**Age (years) [mean ± SD]**	38.41 ± 16.40	42.95 ± 16.68	< 0.001	38.18 ± 1630	43.18 ± 16.72	< 0.001
**Male n (%)**	2479 (44.30)	2550 (45.60)	0.177	2468 (44.10)	2561 (45.80)	0.077
**Educational status**						
under college n (%)	2452 (43.80)	3032 (54.20)	< 0.001	2440 (43.60)	3044 (54.40)	< 0.001
bachelor n (%)	2979 (53.20)	2387 (42.70)	< 0.001	2990 (53.40)	2376 (42.50)	< 0.001
master or doctor n (%)	165 (3.00)	177 (3.10)	< 0.001	166 (3.00)	176 (3.10)	< 0.001
**Body mass index [mean ± SD] (kg/m** ^**2**^ **)**	23.01 ± 3.45	23.48 ± 3.30	< 0.001	23.01 ± 3.47	23.48 ± 3.28	< 0.001
**Obesity n (%)**	2014 (36.00)	2268 (40.50)	< 0.001	2021 (36.10)	2261 (40.40)	< 0.001
**Current smoker n (%)**	1006 (18.00)	1016 (18.20)	0.805	1011 (18.10)	1011 (18.10)	1
**Alcohol use n (%)**	916 (16.40)	734 (13.10)	< 0.001	911 (16.30)	739 (13.20)	< 0.001
**Work intensity**						
light, n (%)	3171 (56.60)	2928 (52.30)	< 0.001	3160 (56.50)	2939 (52.50)	< 0.001
moderate, n (%)	1788 (32.00)	1491 (26.70)	< 0.001	1800 (32.10)	1479 (26.40)	< 0.001
heavy, n (%)	637 (11.40)	1177 (21.00)	< 0.001	636 (11.40)	1178 (21.10)	< 0.001
**Hyperlipidemia n (%)**	226 (4.00)	192 (3.40)	0.09	226 (4.00)	192 (3.40)	0.09
**Hypertension n (%)**	341 (6.10)	332 (5.90)	0.72	332 (5.90)	341 (6.10)	0.72

### Association of dietary glycine, histidine, and glycine + histidine with hyperlipidemia and hypertension

After adjusting for potential variables, dietary glycine, histidine, and glycine + histidine showed an inverse association with hyperlipidemia and hypertension. Compared to participants in the 1st and 2nd tertiles, dietary glycine exhibited an inverse association with hyperlipidemia and hypertension; the OR (95% CIs) for those in the 3rd tertile of hyperlipidemia and hypertension was 0.64 (0.49–0.84) (*p* = 0.003) and 0.70 (0.56–0.88) (*p* = 0.001), respectively. In participants with dietary histidine intake, compared to the 1st and 2nd tertiles, the OR (95% CIs) for those in the 3rd tertile of hyperlipidemia and hypertension was 0.63 (0.49–0.82) (*p* = 0.002) and 0.80 (0.64–0.99) (*p* = 0.025), respectively. In participants with dietary glycine + histidine intake, the OR (95% CIs) for those in the 3rd tertile of hyperlipidemia and hypertension, compared to the 1st and 2nd tertiles, was 0.64 (0.49–0.83) (*p* = 0.003) and 0.74 (0.59–0.92) (*p* = 0.004), respectively (Table 2).

**Table 2:** ORs (95% CIs) for hyperlipidemia and hypertension based on the tertiles of dietary glycine, histidine, and glycine + histidine in the NHAP, 2014–2019

Glycine	Tertile 1	Tertile 2	Tertile 3	*P* for trend
**All hyperlipidemia (cases/** ***n*** **)**	**138/418**	**167/418**	**113/418**	
Model 1	1	0.93 (0.73–1.18)	0.61 (0.47–0.79)	< 0.001
Model 2	1	1.03 (0.81–1.31)	0.69 (0.53–0.90)	0.007
Model 3	1	1.02 (0.80–1.31)	0.64 (0.49–0.84)	0.003
**All hypertension (cases/n)**	**197/673**	**282/673**	**194/673**	
Model 1	1	0.99 (0.81–1.21)	0.65 (0.53–0.81)	< 0.001
Model 2	1	1.05 (0.85–1.28)	0.71 (0.57–0.88)	0.001
Model 3	1	1.08 (0.88–1.33)	0.70 (0.56–0.88)	0.001
**Histidine**				
**All hyperlipidemia (cases/n)**	**147/418**	**145/418**	**126/418**	
Model 1	1	0.76 (0.60–0.97)	0.61 (0.47–0.78)	< 0.001
Model 2	1	0.82 (0.64–1.05)	0.69 (0.54–0.89)	0.005
Model 3	1	0.86 (0.67–1.10)	0.63 (0.49–0.82)	0.002
**All hypertension (cases/n)**	**191/673**	**257/673**	**225/673**	
Model 1	1	0.95 (0.78–1.18)	0.74 (0.60–0.92)	0.004
Model 2	1	1.01 (0.82–1.25)	0.81 (0.65-1.00)	0.034
Model 3	1	1.08 (0.87–1.34)	0.80 (0.64–0.99)	0.025
**Glycine + Histidine**				
**All hyperlipidemia (cases/n)**	**142/418**	**157/418**	**119/418**	
Model 1	1	0.84 (0.66–1.07)	0.61 (0.48–0.79)	< 0.001
Model 2	1	0.93 (0.73–1.19)	0.70 (0.54–0.90)	0.006
Model 3	1	0.95 (0.74–1.22)	0.64 (0.49–0.83)	0.003
**All hypertension (cases/n)**	**195/673**	**272/673**	**206/673**	
Model 1	1	0.96 (0.78–1.18)	0.69 (0.56–0.85)	< 0.001
Model 2	1	1.02 (0.83–1.25)	0.75 (0.60–0.93)	0.006
Model 3	1	1.07 (0.87–1.32)	0.74 (0.59–0.92)	0.004

Model 1 was adjusted for age and gender. Model 2 was adjusted for the variables in Model 1 plus alcohol consumption, smoking status, and work intensity. Model 3 included adjustments for the variables in Model 2 as well as educational status, monthly income, and BMI

### The association of high glycine and high histidine (HGHH) group with hyperlipidemia and hypertension

**Fig. 2:**
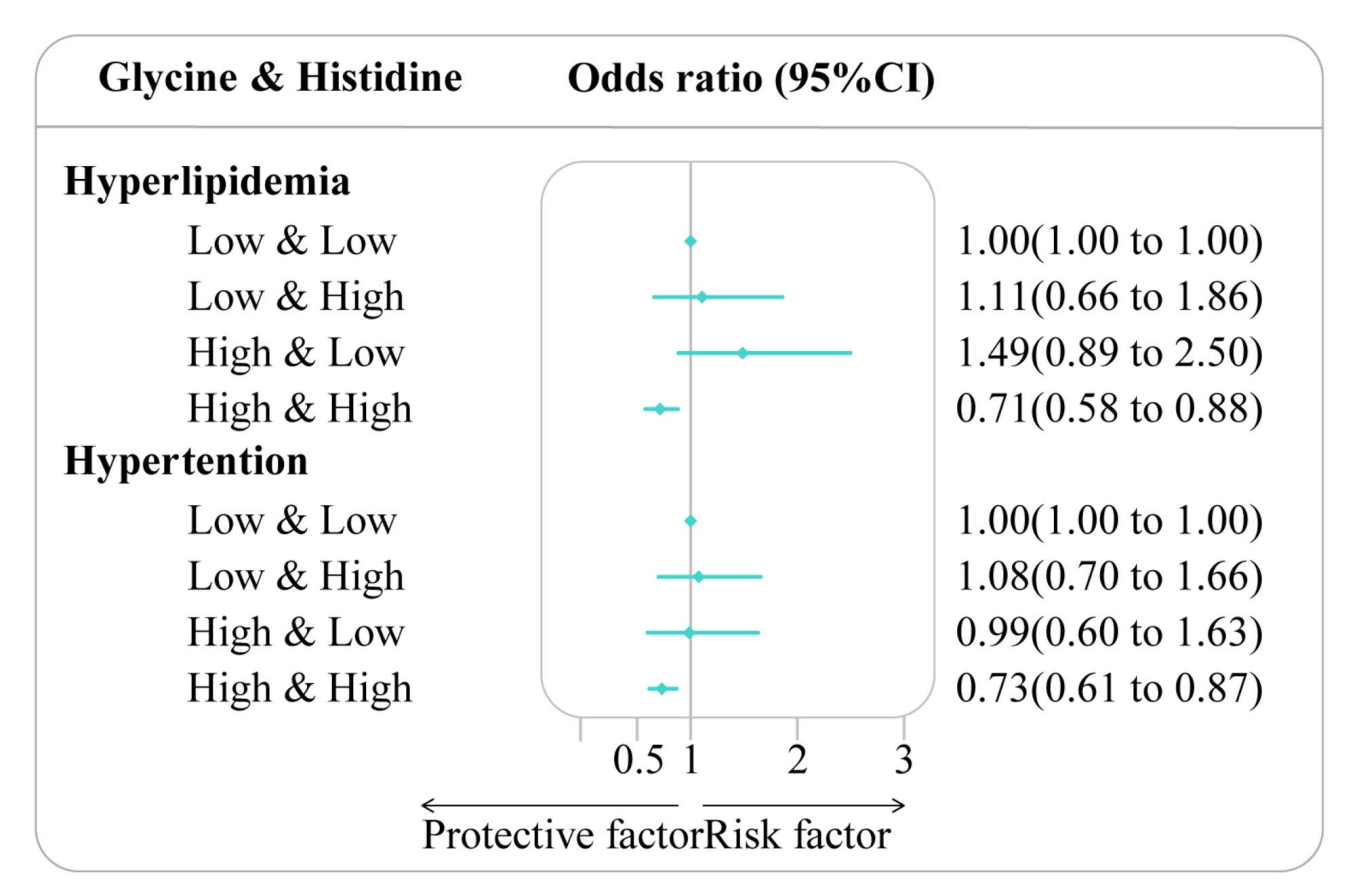
The ORs (95% CIs) for hyperlipidemia and hypertension by high dietary glycine and high histidine (HGHG) group. Association between high glycine and high histidine group and hyperlipidemia and hypertension among adults in the Nutrition Health Atlas Project, 2014–2019. Point estimates represent the ORs and horizontal lines indicate the 95% CIs. ORs: Odd ratios; CIs: confidence intervals

In the context of elevated levels of glycine and histidine, a statistically significant association was observed between high dietary glycine and high histidine (HGHH) group and the occurrence of hyperlipidemia and hypertension. For dietary glycine and histidine, compared with participants in the LGLH group, the risk of hyperlipidemia and hypertension decreased in the high glycine and high histidine level group; the OR (95% CIs) were 0.71 (0.58–0.88) (*p* < 0.01) and 0.73 (0.61–0.87) (*p* < 0.01), respectively (Fig. 2).

### Subgroup analysis

**Fig. 3:**
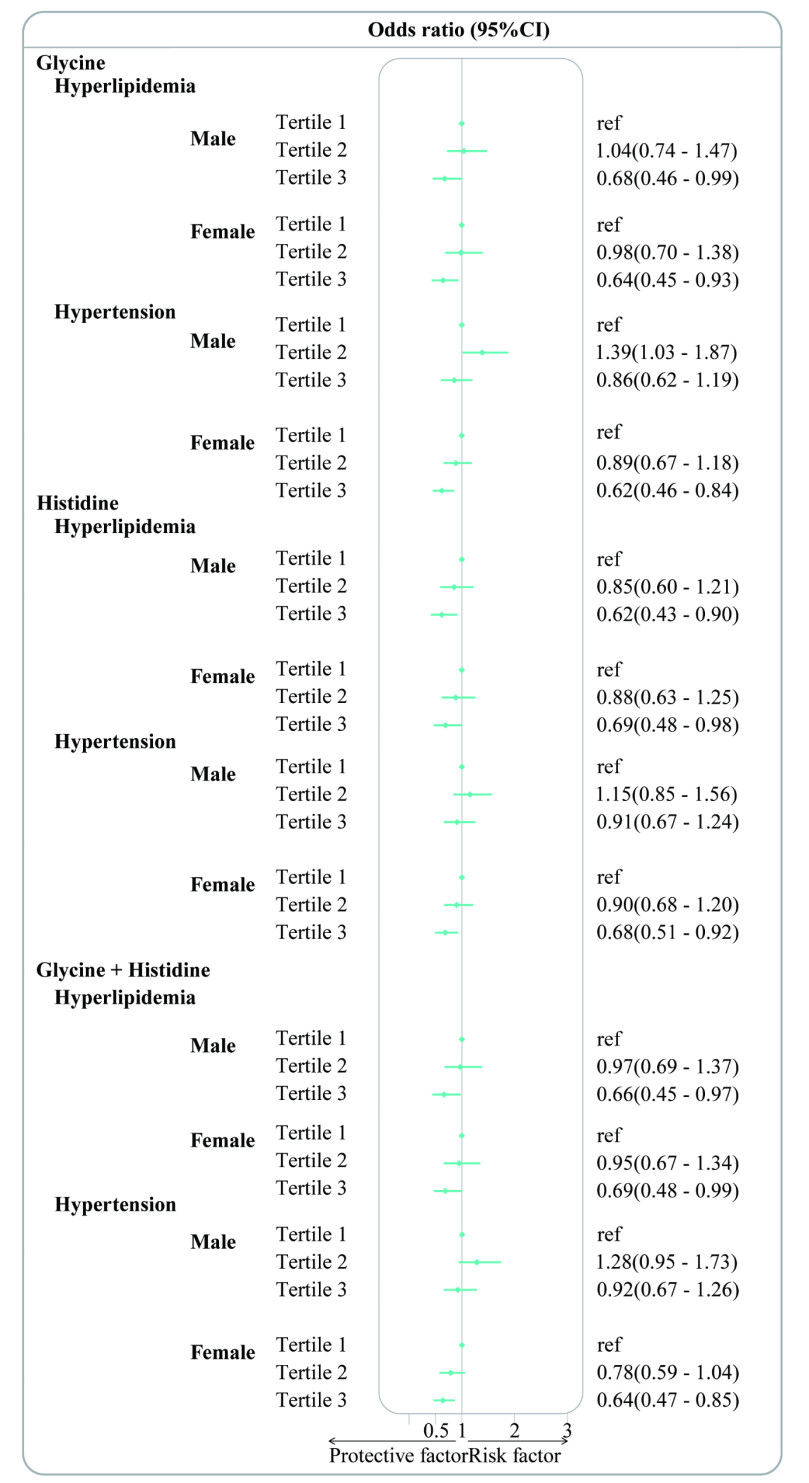
Subgroup analysis based on gender (male and female). Association of dietary glycine, histidine, and glycine + histidine with hyperlipidemia and hypertension among adults in the Nutrition Health Atlas Project, 2014–2019. Point estimates represent the ORs and horizontal lines indicate the 95% CIs. ORs: Odd ratios; CIs: confidence intervals

**Fig. 4:**
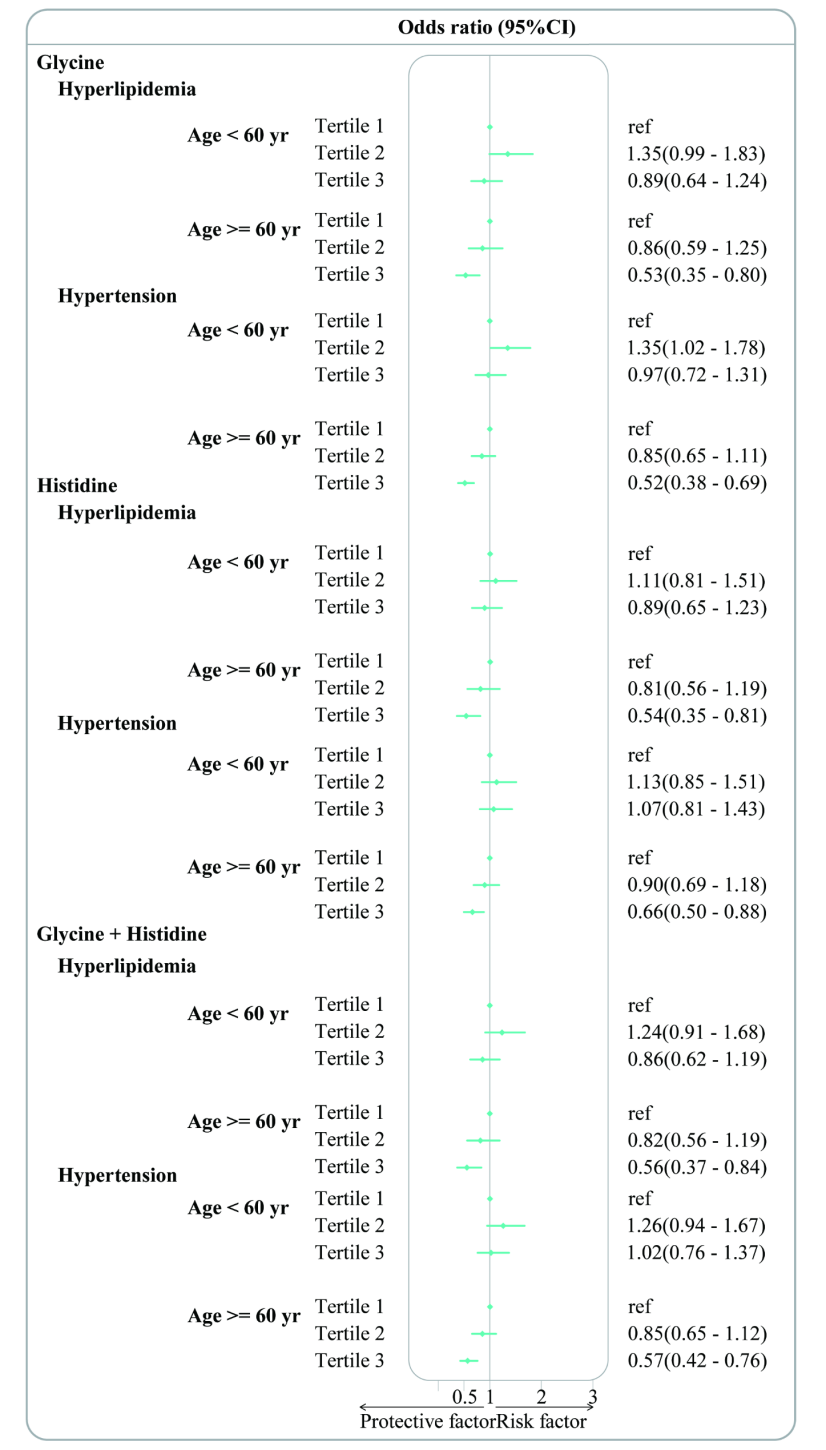
Subgroup analysis based on age groups (aged < 60 and ≥ 60 years). Association of dietary glycine, histidine, and glycine + histidine with hyperlipidemia and hypertension among adults in the Nutrition Health Atlas Project, 2014–2019. Point estimates represent the ORs and horizontal lines indicate the 95% CIs. ORs: Odd ratios; CIs: confidence intervals

**Fig. 5:**
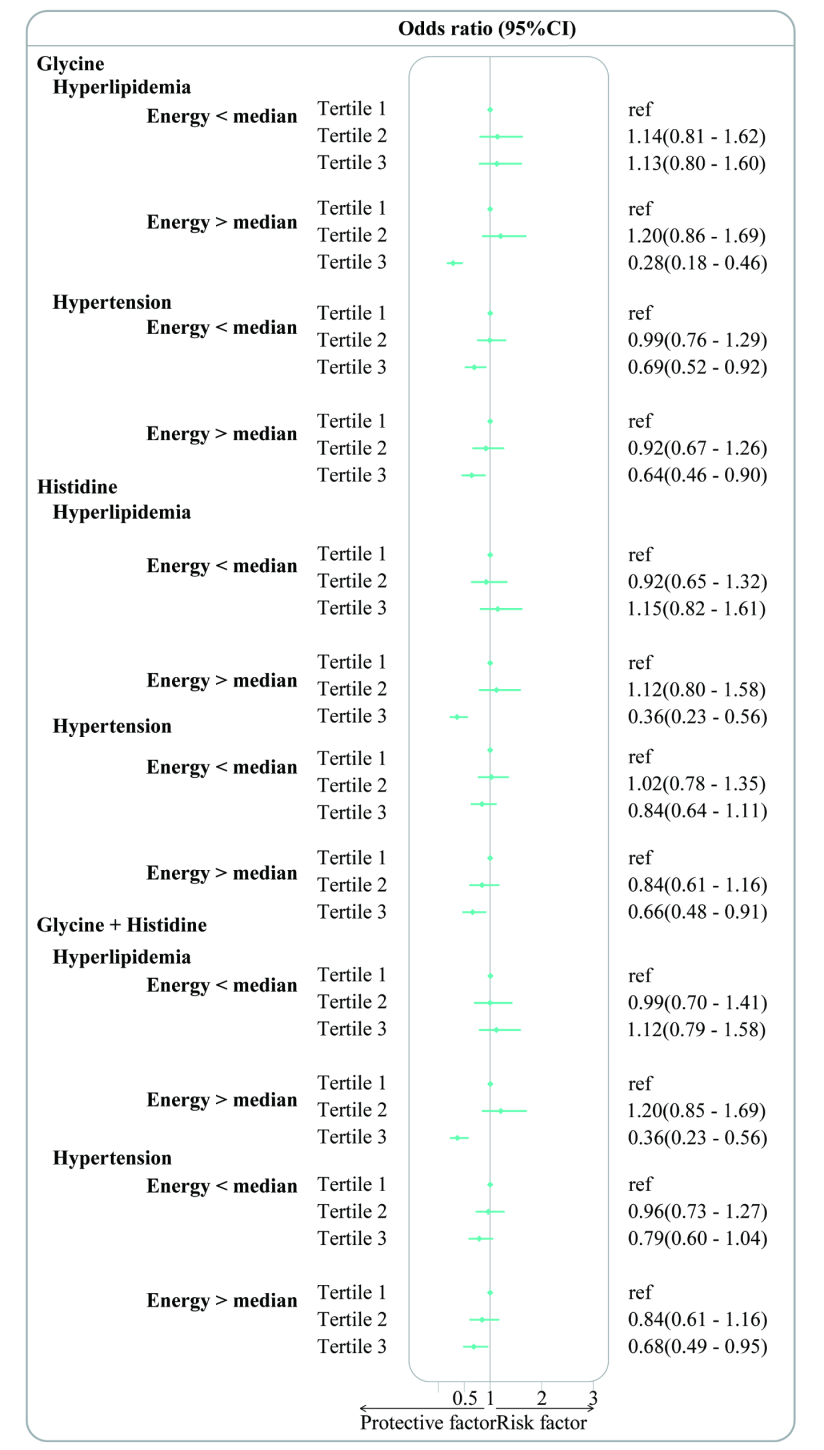
Subgroup analysis based on median level of energy intake (energy intake < median > energy intake). Association of dietary glycine, histidine, and glycine + histidine with hyperlipidemia and hypertension among adults in the Nutrition Health Atlas Project, 2014–2019. Point estimates represent the ORs and horizontal lines indicate the 95% CIs. ORs: Odd ratios; CIs: confidence intervals

An inverse relationship was observed in the analysis conducted by gender (male, female), age groups (< 60 years, ≥ 60 years), median energy intake (energy < median, energy > median). Gender-based analysis revealed a significant association between dietary glycine and both hyperlipidemia and hypertension in female participants. Compared to participants in the 1st and 2nd tertiles, the OR (95% CIs) for those in the 3rd tertile for hyperlipidemia and hypertension were 0.64 (0.45–0.93) (*p* < 0.05) and 0.62 (0.46–0.84) (*p* < 0.01), respectively. Dietary histidine intake was found to be significantly associated with hyperlipidemia in male participants and hypertension in female participants. Compared to the 1st and 2nd tertiles OR (95% CIs) for those in the 3rd tertile of dietary histidine for hyperlipidemia, was 0.62 (0.43–0.90) (*p* < 0.05) in males and 0.68 (0.51–0.92) (*p* < 0.05) in females, respectively. Dietary glycine + histidine was significantly associated with hypertension in female participants. The OR (95% CIs) for those in the 3rd tertile of dietary glycine + histidine, compared to the 1st and 2nd tertiles, was 0.64 (0.47–0.85) (*p* < 0.05) for females (Fig. 3). Based on the age groups, participants aged ≥ 60 years with dietary glycine showed a significant association with hyperlipidemia and hypertension. Compared to the 1st and 2nd tertiles OR (95% CIs), those in the 3rd tertile of dietary glycine for hyperlipidemia and hypertension were 0.53 (0.35–0.80) (*p* < 0.05) and 0.52 (0.38–0.69) (*p* < 0.001), respectively. In participants with dietary histidine intake aged ≥ 60 years, the OR (95% CIs) for hypertension and hyperlipidemia in the 3rd tertile compared to the 1st and 2nd tertiles were 0.54 (0.35–0.81) (*p* < 0.05) and 0.66 (0.50–0.88) (*p* < 0.05), respectively. Dietary glycine + histidine was similarly significant associated with hyperlipidemia and hypertension in participants aged ≥ 60 years. The OR (95% CIs) for hyperlipidemia and hypertension in the 3rd tertile compared to the 1st and 2nd tertiles of dietary glycine + histidine were 0.56 (0.37–0.84) (*p* < 0.05) and 0.57 (0.42–0.76) (*p* < 0.001), respectively (Fig. 4). A significant association was observed when energy intake exceeded the median level. In participants with dietary glycine, compared to the 1st and 2nd tertiles, the OR (95% CI) for hyperlipidemia in the 3rd tertile of dietary glycine was 0.28 (0.18–0.46) (*p* < 0.001) among those with an energy intake exceeding the median level. Compared to participants in the 1st and 2nd tertiles, those in the 3rd tertile of dietary glycine for hypertension had an OR (95% CI) of 0.69 (0.52–0.92) (*p* < 0.01) for those with energy intake above the median level, and 0.64 (0.46–0.90) (*p* < 0.05) for those with energy intake below the median level. Dietary histidine was significantly associated with hyperlipidemia and hypertension in participants with energy intake greater than the median energy level. For hyperlipidemia and hypertension, compared to participants in the 1st and 2nd tertiles of dietary histidine, the OR (95% CIs) for those in the 3rd tertile were 0.36 (0.23–0.56) (*p* < 0.001) and 0.66 (0.48–0.91) (*p* < 0.05), respectively. Dietary glycine + histidine was significantly associated with hyperlipidemia in participants with energy intake greater than the median level. Compared to individuals in the 1st and 2nd tertiles of dietary glycine + histidine for hyperlipidemia, the OR (95% CI) for those in the 3rd tertile was 0.36 (0.23–0.56) (*p* < 0.001) (Fig. 5).

### Sensitivity analysis

On the basis of participant’s non-smoker status, a significant inverse association was observed between dietary intake of glycine, histidine, and glycine + histidine, and hyperlipidemia and hypertension. Compared to participants in the 1st and 2nd tertiles, the OR (95% CI) for those in the 3rd tertile of glycine for hyperlipidemia and hypertension were 0.70 (0.51–0.96) (*p* < 0.05) and 0.63 (0.48–0.82) (*p* < 0.001); for histidine, 0.71 (0.53–0.96) (*p* < 0.05) and 0.77 (0.60-1.00) (*p* < 0.05); and for glycine + histidine, 0.68 (0.50–0.93) (*p* < 0.05) and 0.66 (0.51–0.86) (*p* < 0.05), respectively (Table S1). A significant inverse relationship was found based on the individuals’ non-drinking status. Compared to participants in the 1st and 2nd tertiles, the OR (95% CI) for those in the 3rd tertile of glycine for hyperlipidemia and hypertension were 0.64 (0.46–0.87) (*p* < 0.05) and 0.62 (0.48–0.80) (*p* < 0.001); for histidine, 0.66 (0.49–0.90) (*p* < 0.05) and 0.73 (0.57–0.94) (*p* < 0.05); and for glycine + histidine, 0.64 (0.47–0.87) (*p* < 0.05) and 0.66 (0.51–0.85) (*p* < 0.001), respectively (Table S2).

## Discussion

This study observed a negative association between dietary intake of energy-adjusted glycine, histidine, and glycine + histidine and hyperlipidemia and hypertension. The HGHH group has been shown to exhibit an inverse relationship with hyperlipidemia and hypertension.

As it is known, in contrast to non-essential amino acids, the body cannot produce essential amino acids. Therefore, dietary protein intake largely determines the levels of these amino acids in the body [2]. Amino acids are recognized to have a crucial role in protein synthesis, muscle cell regeneration, and lipid homeostasis [35]. The results of our investigation demonstrated that glycine consumption in the diet has a counteracting association with hyperlipidemia. These findings are supported by several previous studies. Existing studies suggest that glycine may regulate lipid metabolism [36]. Out of all the amino acids, glycine is the most effective lipid-lowering amino acid and efficiently suppresses cellular very low-density lipoprotein uptake and TG biosynthesis rates; glycine deficiency worsens hypercholesterolemia and atherosclerosis [37–39]. Several other studies have corroborated the association between the plasma cholesterol-lowering effect and glycine. However, the mechanism through which dietary glycine reduces plasma cholesterol levels remains unknown, despite glycine being known to participate in the conjugation of bile acids in the liver, which could potentially increase cholesterol excretion [4, 40]. Additionally, an animal study found that glycine decreased visceral obesity by oxidizing free fatty acids in adipose cells [41, 42]. The findings of our study align with previous studies, indicating that there is a negative relationship between dietary glycine and hyperlipidemia. This suggests that glycine is crucial for lipid metabolism. However, the effects of glycine on lipid profiles in humans have not been sufficiently investigated, and further studies are needed to confirm this association.

Histidine, in addition to glycine, was found to have a protective effect against hyperlipidemia. A study conducted by Menon, Kirthi, et al. concluded that histidine-containing dipeptide supplementation is associated with lower total cholesterol and TG levels [17]. Several other studies have found that histidine has specific antioxidant activities such as inhibiting free radicals and chelating divalent metal ions, reducing triglyceride accumulation in the organs of diabetic rats [43]. The histidine lipid lowering effect is supported by animal studies; two rodent studies found that carnosine and histidine supplementation reduced cholesterol and triglyceride levels (carnosine or histidine 1 g/L for 4 weeks and carnosine or histidine 1 g/L for 8 weeks), respectively [43, 44]. Similarly, a study in which middle-aged women with obesity and a diagnosis of metabolic syndrome received 12 weeks of supplemental histidine (4 g, daily) or a similar placebo found striking results [45]. Consistent with the safeguarding role of histidine in lipid metabolism, our findings on the inverse association between dietary histidine and hyperlipidemia are supported by evidence from both human and animal studies.

The relationship between dietary amino acids and blood pressure in humans has not been extensively studied. Recent studies suggest that inadequate protein intake may cause a deficiency of essential amino acids and a subsequent elevation in blood pressure [46]. Some studies have shown that high dietary protein may reduce high blood pressure [25, 47]. Our study revealed that a higher dietary glycine intake was associated with a lower risk of hypertension, indicating a negative correlation between dietary glycine and high blood pressure. Both cross-sectional [48] and prospective studies [49] have provided evidence supporting the possible preventive effect of glycine in preventing hypertension. A proposed mechanism by which glycine may lower blood pressure involves glycine-gated chloride channels on the endothelium surface; these channels are thought to reduce blood pressure by increasing membrane polarization and producing nitric oxide, a well-known vasodilator [50]. Notably, our findings demonstrate a potential negative association between high blood pressure and glycine intake in a large-sample cross-sectional epidemiological setting.

Furthermore, recent investigations revealed that a substantial intake of dietary histidine exhibits a protective effect against high blood pressure [51]. A study found that histidine could help control blood pressure because it acts as a precursor to nitric oxide in the brain’s vasomotor centres. Oral consumption of L-histidine, a byproduct of L-carnosine degradation, effectively suppressed blood pressure. The hypotensive properties of L-histidine were facilitated by thioperamide, a central H3 receptor antagonist [16]. Our findings consistently showed a concordant inverse association between dietary intake of histidine and hypertension. In this study, the findings on the association between higher levels of dietary glycine and histidine intake and the development of hyperlipidemia and hypertension were consistent with the conclusions of the majority of studies.

Based on the study findings, participants in the HGHH group were associated with a reduced likelihood of developing hyperlipidemia and hypertension. A study has confirmed the efficacy of the free plasma amino acid profile as a marker for evaluating the risk of metabolic disorders, including hyperlipidemia, hypertension, and MetS [35, 52]. Moreover, the use of glycine has been shown to lower hypertension in people with metabolic syndrome [53]. Ntzouvani, A. et al. have found that glycine was inversely correlated with established cardio-metabolic risk factors and metabolic syndrome [54]. Other studies conducted in the Chinese population reported associations between higher dietary histidine and a lower prevalence of overweight and obesity, as well as lower BMI, waist circumference, and high blood pressure [31, 45]. Likewise, a study conducted in Korean adults found a significant association between a higher intake of total essential amino acids and a lower prevalence of metabolic disorders, including hyperlipidemia and hypertension [35]. To the best of our knowledge, a groundbreaking finding of this study was the evident and significant negative association between high dietary glycine and high histidine intake and hyperlipidemia and hypertension.

Based on the subgroup and sensitivity analysis results, this study found a significant inverse association of dietary glycine, histidine, and glycine + histidine with hyperlipidemia and hypertension across genders, age groups, median energy intakes, non-smoker, and non-drinker. Some studies consistently reported gender and age differences in dietary glycine and histidine intake, along with their association with metabolic diseases, while other studies did not support these associations [31, 55–57]. In current study, dietary glycine and histidine showed significant associations with hypertension and hyperlipidemia specifically in male participants. The variation in lifestyle and dietary habits between males and females might explain this gender disparity. Men and women often have different approaches to nutrition, physical activity, stress management, and overall health practices. These differences can significantly impact these health outcomes. For instance, men may consume more calorie-dense and protein-rich foods, while women prioritize balanced diets with more fruits and vegetables along with sweets [58, 59]. Furthermore, exercise routines often vary by gender, with some studies indicating that men are more active than women while women [60] might prefer moderate exercises and housework which is positive associated with metabolic markers, triglyceride, and pre-hypertension [61]. Societal expectations and stressors differ between genders which can affect mental health and subsequently influence physical well-being [62]. Collectively, these distinctions in lifestyle choices contribute to the observed differences in health statuses between males and females. A significant negative association was observed in individuals aged ≥ 60 years between dietary glycine, histidine, glycine + histidine, and hyperlipidemia and hypertension. Similar results have been reported in other studies, as well as in a study conducted on the Korean population aged 66 years [55, 63]. Also, higher energy intake, surpassing the median level, showed a significant negative correlation with the dietary glycine, histidine, and glycine + histidine in relation to hyperlipidemia and hypertension. These findings were congruent with those of other investigations [64]. The inverse association of dietary glycine and histidine with hyperlipidemia and hypertension in individuals over 60 years old, who consume more than the median level of these amino acids, non-smoker, and non-drinker can be explained by their roles in supporting vasodilation, reducing inflammation and oxidative stress, improving metabolic regulation and endothelial function, balancing histamine levels, aiding in protein metabolism, and maintaining overall acid-base balance. These effects contribute to better cardiovascular health, thereby lowering the risks of hyperlipidemia and hypertension in older adults [3, 6, 7, 10, 12–14, 65, 66].

Some limitations associated with this study are worth mentioning. Although we fully adjusted for confounding factors, we cannot exclude the potential for residual confounding due to relevant dietary or other variables. Furthermore, this study cannot determine the causative association between dietary glycine and histidine and the development of hyperlipidemia and hypertension. Therefore, interventional studies are needed to confirm this relationship. Additionally, self-reporting may have resulted in an underestimation of the occurrence of several diseases under investigation; for instance, cases of hyperlipidemia and hypertension may not have been accurately reported. Moreover, this study was conducted on a northern Chinese population, necessitating further research to validate this association across the entire Chinese population. Lastly, self-reported lifestyle-related data can lead to misclassification and residual confusion. Finally, although the study revealed a negative relationship between dietary glycine, histidine, and hyperlipidemia and hypertension, further investigation is required to verify this relationship.

## Conclusions

This study evaluated the association of dietary glycine and histidine with hyperlipidemia and hypertension through an Internet-based dietary questionnaire in Chinese. Our findings indicated that dietary glycine and histidine, as well as the high dietary glycine and high histidine groups, revealed an inverse relationship with hyperlipidemia and hypertension. Additionally, the subgroup analysis showed a significant inverse association among males over sixty, individuals with energy intake surpassing the median level, non-smoker and non-drinker.

### Electronic supplementary material

Below is the link to the electronic supplementary material.

Supplementary Material 1

## Data Availability

No datasets were generated or analysed during the current study.
