# Supplementary material for: The inverse associations of glycine and histidine in diet with hyperlipidemia and hypertension
Source: Nutr J. 2024 Aug 22;23:98. doi: 10.1186/s12937-024-01005-4 (PMC11340119; doi:10.1186/s12937-024-01005-4)
Supplement: Supplementary file 1 — Supplementary Material 1 [file 12937_2024_1005_MOESM1_ESM.docx]

**Supplementary Information**

**The inverse associations of glycine and histidine in diet with hyperlipidemia and hypertension**

Mohammad Haroon Rahemi ^1,2†^, Yuting Zhang^1,2†^, Zican Li^1,2†^, Dongwei Guan^1,2^, Defang Li^1,2^, Hongxin Fu^1,2^, Jiaying Yu^1,2^, Junrong Lu^3^, Cheng Wang^4*^, Rennan Feng^1,2*^

1. Department of Nutrition and Food Hygiene, School of Public Health, Harbin Medical University, Heilongjiang, 150081, China.

2. Key Laboratory of Precision Nutrition and Health of Ministry of Education, School of Public Health, Harbin Medical University, Heilongjiang, 150081, China.

3. Department of Interventional Radiology, Harbin Medical University Cancer Hospital, Harbin, 150081, China.

4. Department of Environmental Hygiene, School of Public Health, Harbin Medical University, Heilongjiang, 150081, China.

† These authors contributed equally to this work.

* These authors were co-corresponding authors.

Rennan Feng, E-mail: fengrennan@yeah.net. Department of Nutrition and Food Hygiene, Public Health College, Harbin Medical University,157 Baojian Road, Nan gang District, Harbin, P. R. China, 150086.

Tel: + 86 045187502885; Fax: +86 045187502881.

Cheng Wang, E-mail: wangchenghlj@163.com. Department of Environmental Hygiene, Public Health College, Harbin Medical University, Harbin, P. R. China, 150086.

Tel: + 86 18845166185.

**Supplementary Tables and Figures**

**Table S1.** ORs (95% CIs) for hyperlipidemia and hypertension based on the tertiles of dietary glycine, histidine, and glycine + histidine in the NHAP non-smokers, 2014–2019.

**Table S2.** ORs (95% CIs) for hyperlipidemia and hypertension based on the tertiles of dietary glycine, histidine, and glycine + histidine in the NHAP non-drinkers, 2014–2019.

| **Table S1.** ORs (95% CIs) for hyperlipidemia and hypertension based on the tertiles of dietary glycine, histidine, and glycine + histidine in the NHAP non-smokers, 2014–2019. | | | | |
| --- | --- | --- | --- | --- |
| **Glycine** | **Tertile 1** | **Tertile 2** | **Tertile 3** | **p for trend** |
|  |  |  |  |  |
| **All hyperlipidemia (cases/n)** | **138/418** | **167/418** | **113/418** |  |
| Model 1 | 1 | 1.00 (0.75-1.32) | 0.67 (0.49-0.91) | 0.007 |
| Model 2 | 1 | 1.04 (0.78-1.38) | 0.73 (0.53-0.99) | 0.031 |
| Model 3 | 1 | 1.07 (0.80-1.43) | 0.70 (0.51-0.96) | 0.018 |
| **All hypertension (cases/n)** | **197/673** | **282/673** | **194/673** |  |
| Model 1 | 1 | 0.99 (0.79-1.26) | 0.59 (0.46-0.77) | < 0.001 |
| Model 2 | 1 | 1.02 (0.80-1.29) | 0.63 (0.48-0.81) | < 0.001 |
| Model 3 | 1 | 1.07 (0.84-1.36) | 0.63 (0.48-0.82) | < 0.001 |
| **Histidine** |  |  |  |  |
| **All hyperlipidemia (cases/n)** | **147/418** | **145/418** | **126/418** |  |
| Model 1 | 1 | 0.80 (0.61-1.07) | 0.68 (0.51-0.92) | 0.012 |
| Model 2 | 1 | 0.83 (0.63-1.11) | 0.74 (0.55-1.00) | 0.054 |
| Model 3 | 1 | 0.88 (0.66-1.19) | 0.71 (0.53-0.96) | 0.026 |
| **All hypertension (cases/n)** | **191/673** | **257/673** | **225/673** |  |
| Model 1 | 1 | 0.94 (0.74-1.19) | 0.72 (0.56-0.92) | 0.006 |
| Model 2 | 1 | 0.97 (0.76-1.23) | 0.76 (0.59-0.98) | 0.024 |
| Model 3 | 1 | 1.04 (0.82-1.34) | 0.77 (0.60-1.00) | 0.026 |
| **Glycine + Histidine** |  |  |  |  |
| **All hyperlipidemia (cases/n)** | **142/418** | **157/418** | **119/418** |  |
| Model 1 | 1 | 0.89 (0.67-1.17) | 0.66 (0.49-0.89) | 0.005 |
| Model 2 | 1 | 0.93 (0.70-1.23) | 0.72 (0.53-0.97) | 0.026 |
| Model 3 | 1 | 0.98 (0.73-1.31) | 0.68 (0.50-0.93) | 0.012 |
| **All hypertension (cases/n)** | **195/673** | **272/673** | **206/673** |  |
| Model 1 | 1 | 0.95 (0.75-1.20) | 0.62 (0.48-0.81) | < 0.001 |
| Model 2 | 1 | 0.98 (0.77-1.25) | 0.66 (0.59-0.86) | 0.001 |
| Model 3 | 1 | 1.06 (0.83-1.35) | 0.66 (0.51-0.86) | 0.001 |
| Model 1 was adjusted for age and gender. Model 2 was adjusted for the variables in Model 1 plus alcohol consumption, and work intensity. Model 3 included adjustments for the variables in Model 2 as well as educational status, monthly income, and BMI. | | | | |

| **Table S2.** ORs (95% CIs) for hyperlipidemia and hypertension based on the tertiles of dietary glycine, histidine, and glycine + histidine in the NHAP non-drinkers, 2014–2019. | | | | |
| --- | --- | --- | --- | --- |
| **Glycine** | **Tertile 1** | **Tertile 2** | **Tertile 3** | **p for trend** |
|  |  |  |  |  |
| **All hyperlipidemia (cases/n)** | **138/418** | **167/418** | **113/418** |  |
| Model 1 | 1 | 0.95 (0.72-1.26) | 0.62 (0.46-0.84) | 0.002 |
| Model 2 | 1 | 0.98 (0.74-1.31) | 0.65 (0.48-0.89) | 0.005 |
| Model 3 | 1 | 1.00 (0.75-1.34) | 0.64 (0.46-0.87) | 0.004 |
| **All hypertension (cases/n)** | **197/673** | **282/673** | **194/673** |  |
| Model 1 | 1 | 1.01 (0.81-1.28) | 0.60 (0.46-0.77) | < 0.001 |
| Model 2 | 1 | 1.03 (0.82-1.30) | 0.61 (0.47-0.78) | < 0.001 |
| Model 3 | 1 | 1.09 (0.86-1.38) | 0.62 (0.48-0.80) | < 0.001 |
| **Histidine** |  |  |  |  |
| **All hyperlipidemia (cases/n)** | **147/418** | **145/418** | **126/418** |  |
| Model 1 | 1 | 0.81 (0.61-1.08) | 0.64 (0.48-0.86) | 0.003 |
| Model 2 | 1 | 0.83 (0.62-1.10) | 0.68 (0.50-0.91) | 0.01 |
| Model 3 | 1 | 0.86 (0.64-1.15) | 0.66 (0.49-0.90) | 0.008 |
| **All hypertension (cases/n)** | **191/673** | **257/673** | **225/673** |  |
| Model 1 | 1 | 0.02 (0.81-1.30) | 0.70 (0.55-0.90) | 0.002 |
| Model 2 | 1 | 1.03 (0.81-1.31) | 0.71 (0.55-0.91) | 0.003 |
| Model 3 | 1 | 1.11 (0.87-1.42) | 0.73 (0.57-0.94) | 0.006 |
| **Glycine + Histidine** |  |  |  |  |
| **All hyperlipidemia (cases/n)** | **142/418** | **157/418** | **119/418** |  |
| Model 1 | 1 | 0.87 (0.65-1.15) | 0.62 (0.46-0.84) | 0.002 |
| Model 2 | 1 | 0.90 (0.68-1.19) | 0.65 (0.48-0.88) | 0.005 |
| Model 3 | 1 | 0.93 (0.69-1.25) | 0.64 (0.47-0.87) | 0.004 |
| **All hypertension (cases/n)** | **195/673** | **272/673** | **206/673** |  |
| Model 1 | 1 | 1.01 (0.80-1.27) | 0.63 (0.49-0.81) | < 0.001 |
| Model 2 | 1 | 1.02 (0.81-1.29) | 0.64 (0.50-0.83) | < 0.001 |
| Model 3 | 1 | 1.10 (0.87-1.40) | 0.66 (0.51-0.85) | < 0.001 |
| Model 1 was adjusted for age and gender. Model 2 was adjusted for the variables in Model 1 plus smoking status, and work intensity. Model 3 included adjustments for the variables in Model 2 as well as educational status, monthly income, and BMI. | | | | |
